# Evolution of an increased performance under acute challenge does not exacerbate vulnerability to chronic stress

**DOI:** 10.1038/s41598-022-06060-7

**Published:** 2022-02-08

**Authors:** Małgorzata M. Lipowska, Edyta T. Sadowska, Rupert Palme, Paweł Koteja

**Affiliations:** 1 Institute of Environmental Sciences, Jagiellonian University, Kraków, Poland; 2 Department of Biomedical Sciences, University of Veterinary Medicine, Vienna, Austria

**Keywords:** Experimental evolution, Metabolism

## Abstract

An adequate stress response plays a vital role in coping with challenges. However, if selection for improved coping with an acute challenge affects the entire stress response system, susceptibility to adverse effects of chronic stressors can be deepened. Here, we used bank voles from lines selected for high swim-induced aerobic metabolism (A) and unselected control (C), and asked if the selection affected sensitivity to chronic mild stress (CMS). The voles were first habituated to daily weighing and feces collection for three weeks, and then for two weeks were exposed to CMS or remained undisturbed. The habituation itself resulted in an increased swim-induced oxygen consumption in both line types, and a decreased body mass. The CMS treatment caused reduction of food consumption in the second week of the experiment, and, in males, a decline in the metabolic rate. Paradoxically, fecal corticosterone metabolites decreased in the CMS-treated group. The response to CMS did not differ between the line types. Thus, the selection for increased performance was not traded off by increased vulnerability to chronic stress. The counter-intuitive results may even lead to a speculation that bank voles—and perhaps also other animals—prefer experiencing unpredictable, unpleasant stressors over the monotony of standard laboratory housing.

## Introduction

Despite the popularity of studies concerning chronic stress and its effects on animals and humans, the subject is still elusive^1–3^. An ability to perceive and adequately respond to an acute stressor helps animals to cope with challenges. However, if evolution of improved coping with a particular challenge involves changes in the entire stress response system, sensitivity to other stressors, and particularly to a chronic exposure to even mild stressors, can be also affected. Consequently, health and performance under the chronic stress conditions could be compromised. Here, we used an experimental evolution approach to ask whether an increased aerobic exercise performance attained in an acute swimming trial is traded off by an increased susceptibility to chronic stress.

A brief stress may temporarily improve an animal’s performance and coping with the stressing challenge, but chronic stress is considered detrimental^4,5^. Common outcomes include feeding disorders leading to anorexia or adiposity^6–8^, behavioral disorders such as anxiety or helpless passivity^1,9–11^, and circulatory system malfunctions^12,13^. Because of these symptoms’ similarity to those characterizing depression in humans, chronic stress has been often applied in laboratory animals to provide models of depression^8,14,15^. Although the exact protocols vary among studies, they usually follow the scheme of chronic mild stress (CMS) protocol. The protocol involves a persistent exposure to a series of relatively mild stressors, such as circadian rhythm disruption, intermittent noise or vibration, temporary deficiency of food or water, or non-favorable housing conditions, appearing in an unpredictable order for a period ranging from days to months^14–16^. However, as the effects of CMS protocols vary greatly among studies, its universality is subject to discussion^1,8,16^.

Another question is whether chronic exposure to mild stressors is indeed as unfavorable as assumed. Free-living animals frequently encounter cues of threat, and experience hunger, thirst or harsh weather. Despite this, we do not consider natural conditions as chronically stressful, do not expect the animals to be “depressed”, and since these stressors do not prevent the species from living and thriving, we believe the animals are adapted to such environment^17^. On the other hand, laboratory animals are provided with comfortable, stable, safe conditions with ad libitum access to food and water, and therefore the animals are deprived of most of the stressors they would experience when living in the wild. Such bland conditions, paired with an array of stressors evoked by limited mobility, high population density and continuous human proximity, can induce a state of chronic stress^18,19^. From this perspective, the studies involving CMS procedure do not involve “stressed” and “unstressed” (control) experimental groups, but rather two groups differing in the type of chronic stress they experience. Moreover, the CMS condition could even be perceived by the animals as the less stressful one.

Among the most often studied aspects of the animals’ response to stress is the activity of the hypothalamic–pituitary–adrenal (HPA) axis, and its main effector hormones, glucocorticoids such as corticosterone^20,21^. The strength of the HPA signaling depends on both, the level of the hormones and abundance of the receptors they can bind to^13^. However, because evaluation of the receptor expression is much more difficult and more invasive than measuring levels of the circulating hormones or its excreted metabolites, most studies on stress responses concentrate only on the hormonal element of the signaling pathway.

Glucocorticoids mediate response to both stressful incidents and to circumstances demanding increased metabolic output^22^. A moderate increase in glucocorticoid level is typically associated with promotion of physical activity, resource use and increased metabolic rate, such as observed in metabolically-demanding life history stages^23,24^ or following intense physical activity^25,26^. On the contrary, a strong glucocorticoid response can result in suppression of the activity^22^, which can benefit an animal facing a threat that cannot be escaped from, but can be avoided by hiding and waiting^27^. Therefore, when facing a physically-demanding challenge, a moderate increase in glucocorticoid level can support ability to achieve high physical performance, but this performance can get hindered if the glucocorticoid response to the challenge achieves level associated with promotion of the reactive coping strategy^22,28,29^.

Prolonged or repeated exposure to stressors results in prolonged elevation of glucocorticoid levels, and altered scope of response to further stressors^2^. However, the scope and direction of this effect may depend on the nature of the stressors and vary among species or even individuals. In humans, depression induced by stress or trauma can take two forms, accompanied by a chronically increased or decreased glucocorticoid level^3,7^. Similarly, in rats and mice, the responses vary significantly among strains^6,30^. Particularly, mice from lines selected for high or low HPA reactivity expressed behavior resembling distinct types of human depression^31,32^.

Because glucocorticoids play a role in modulation of metabolic rate and physical activity^13,22^, disruption of their level may hinder the animal’s performance in a challenge requiring physical effort. In depression-oriented studies, such a challenge is often modelled with a swimming trial, which involves placing an animal on a water surface and measuring the time it spends on active swimming^33^. The effort it puts into swimming is interpreted as a measure of animal’s self-preservation struggle, the decrease of which is associated with depression-like state^33^. As the animals need little energy to stay afloat, the physical activity is not externally forced, but is internally motivated by a stress response to the unfavorable environment and willingness to escape from it.

This study is based on a unique experimental evolution model, with lines of a non-laboratory rodent, the bank vole (*Myodes glareolus*), selected for high rate of aerobic metabolism achieved during swimming (A lines)^34–36^. The selection test is similar to the swimming trial applied in the studies on depression^33^, but instead of observing the animal’s behavior we measure its metabolic rate – a value directly associated with the intensity of a swimming effort. After 25 generations, animals from the A lines achieve over 70% higher maximum swim-induced metabolic rate (VO_2_swim) than those from unselected, control (C) lines. The selection has also increased the forced-running maximum metabolic rate by about 30%^37^. Voles from the A lines spend more time on active swimming, and, unlike the C-line voles, during the swimming trial work up to their physiological performance limit they achieve during running^37,38^. Additionally, A-line animals exhibit increased activity and escape propensity in an open field test^39^. Therefore, in these lines the animals have developed not only an increased metabolic capacity, but also a more proactive response to challenging situations.

In both line types the swimming trial elicits an increase in glucocorticoid level strong enough to hinder the animal’s metabolic performance^28^. Interestingly though, the selection did not affect the baseline or trial-induced corticosterone levels^28^. However, the maximum, pharmacologically-induced corticosterone level was lowered in the A lines^40^. Taken at a face value, the results would suggest that in the A lines the stress associated with the swimming challenge is perceived as relatively more acute, as the glucocorticoid response it evokes is closer to the maximum response. We also observed that two weeks of exposure to CMS elevated baseline corticosterone level in voles from both line types, but the effect was more pronounced in the A than in the C lines (unpublished). Based on those results, we hypothesized that the A-line voles are more susceptible to CMS-induced depression-like states than the C-line ones, and this effect would be reflected in changes of their swimming performance.

However, we realize that the above hypothesis may be oversimplistic. Firstly, it cannot be excluded that selection has also affected expression of glucocorticoid hormone receptors, an effect which would further modulate the strength of glucocorticoid signaling and severity of the stress perceived. Additionally, changes in receptor expression can affect the rate of glucocorticoid levels return to baseline after stress^23^, determining the duration of exposure to elevated hormone levels. Thus, the comparisons of plasma corticosterone level provide only a partial support of the hypothesis. Secondly, we should also consider a possibility that the bank vole, which is not a laboratory species, can be more adapted to functioning in an ever-changing environment than in the comfortable but monotonous laboratory conditions. Captive breeding often involves more or less intentional domestication, which is known to be associated with changes in the HPA axis^41^. However, in guinea pigs kept for 30 generations in captivity, the behavior and the HPA axis activity did not differ from wild-derived animals, and was very distinct from that in domesticated strains^42^. In our bank vole selection experiment care is taken to minimize domestication, and after more than 30 generations in captivity (see Methods), the animals are not nearly as docile as e.g., laboratory mice. Therefore, it is not entirely unlikely that the animals’ performance would *improve*, rather than decline, under CMS conditions, implying the stressful aspect of standard housing conditions.

**Figure 1:**
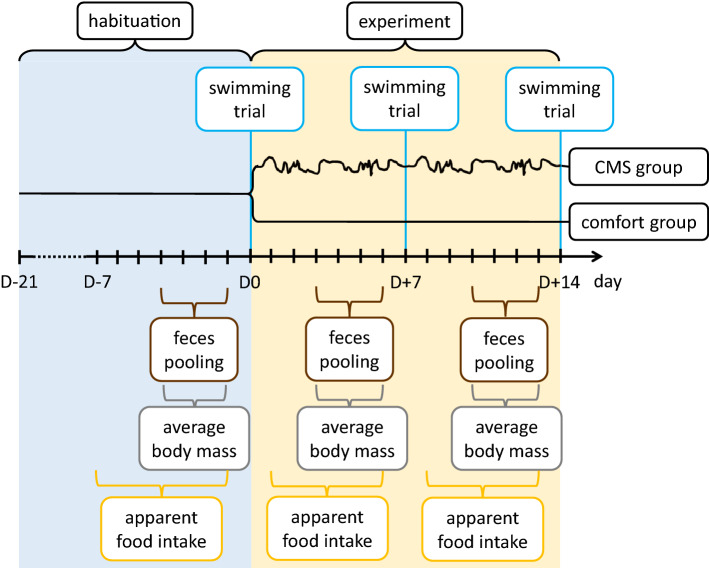
Schematic overview of the experimental design. At days D-21- D0 all animals were undergoing the same habituation procedure, involving daily weighing and a 20-min transfer to an empty cage allowing for feces collection. After the D0 swimming trial the experimental groups were established and the chronic mild stress (CMS) treatment was applied to the CMS group. The CMS treatment included a series of mild, unpredictable stressors (Table 1) and lasted from the night after D0 to the swimming trial at D+14. The daily manipulations introduced during the habituation period continued throughout the experiment, except in the swimming trial days.

Here, we tested the hypothesis that the evolution of intense aerobic exercise performance in the A lines was accompanied by an increased sensitivity to chronic stress. To test the hypothesis, voles from the A and C lines were assigned to groups subjected to two weeks of the CMS treatment or maintained in “comfort” conditions (Table 1, Fig. 1). Body mass, food consumption, corticosterone metabolites in feces, and the swim-induced metabolic rate were measured before, during and after the two-week period. If the hypothesis is true, voles from the A lines should show a larger reduction of swimming performance and a more severe physiological dysregulation (altered food consumption, body mass and corticosterone level) under chronically stressful conditions.

**Table 1:** The daily procedures applied to all animals throughout the 22-day habituation and 14-day experiment, a sequence of stress-inducing procedures applied in the experimental “CMS” group, and swimming trials applied to both “CMS” and “comfort” groups.

Day	Time		Stressor
daily, D-21 to D+13	8:00–11:00	Morning	Habituation procedures: handling, weighing, 20-min translocation to an empty cage for fecal sampling
D0	8:00–18:00	Daytime	transfer to laboratory, 18-min swimming test and new cage
	18:00–8:00	Nighttime & Dawn	CMS treatment starts; 1–2 h periods of light on and off
D+1	8:00–18:00	Daytime	8 h without water bottle
	18:00–8:00	Nighttime & Dawn	15–45 min periods of vibrating cage with 15–30 min periods of rest
D+2	8:00–18:00	Daytime	8 h in new cage with wet bedding
	18:00–8:00	Nighttime & Dawn	light on at night, off at dawn
D+3	8:00–18:00	Daytime	15–45 min periods of vibrating cage with 15–30 min periods of rest
	18:00–8:00	Nighttime & Dawn	15–45 min periods of white noise with 15–30 min periods of rest
D+4	8:00–18:00	Daytime	8 h without water bottle
	18:00–8:00	Nighttime & Dawn	1–2 h periods of light on and off
D+5	8:00–18:00	Daytime	8 h in new cage with no bedding
	18:00–8:00	Nighttime & Dawn	light on at night, off at dawn
D+6	8:00–18:00	Daytime	8 h with no food in feeder
	18:00–8:00	Nighttime & Dawn	15–45 min periods of white noise with 15–30 min periods of rest
D+7	8:00–18:00	Daytime	transfer to laboratory, 18-min swimming test and new cage
	18:00–8:00	Nighttime & Dawn	1–2 h periods of light on and off
D+8	8:00–18:00	Daytime	8 h without water bottle
	18:00–8:00	Nighttime & Dawn	15–45 min periods of vibrating cage with 15–30 min periods of rest
D+9	8:00–18:00	Daytime	8 h in new cage with wet bedding
	18:00–8:00	Nighttime & Dawn	light on at night, off at dawn
D+10	8:00–18:00	Daytime	15–45 min periods of vibrating cage with 15–30 min periods of rest
	18:00–8:00	Nighttime & Dawn	15–45 min periods of white noise with 15–30 min periods of rest
D+11	8:00–18:00	Daytime	8 h without water bottle
	18:00–8:00	Nighttime & Dawn	1–2 h periods of light on and off
D+12	8:00–18:00	Daytime	8 h in new cage with no bedding
	18:00–8:00	Nighttime & Dawn	light on at night, off at dawn
D+13	8:00–18:00	Daytime	8 h with no food in feeder
	18:00–8:00	Nighttime & Dawn	15–45 min periods of white noise with 15–30 min periods of rest
D+14	8:00–18:00	Daytime	transfer to laboratory, 18-min swimming test and new cage

The sessions of handling, weighing and fecal sampling were not performed during the swimming trial days. The stress procedures applied during the daytime usually started immediately after these daily sessions.

## Results

Tables with group composition, complete descriptive statistics and results of LR tests for random effects in the mixed models are presented in Supplement (Supplementary Tables S1–S3). Raw data can be viewed in a Supplementary data file and on scatter plots (left columns of Figs. 3 and 4, and Supplementary Fig. S1). Here we provide the main results of statistical models: significance of the main factors of interest and least squares means with 95% confidence intervals (LSM ± CI), computed for the approximately mean value of the BMavg covariate (22.4 g), the same for all analyses.

### Analyses set 1: effects of selection and habituation

**Figure 2:**
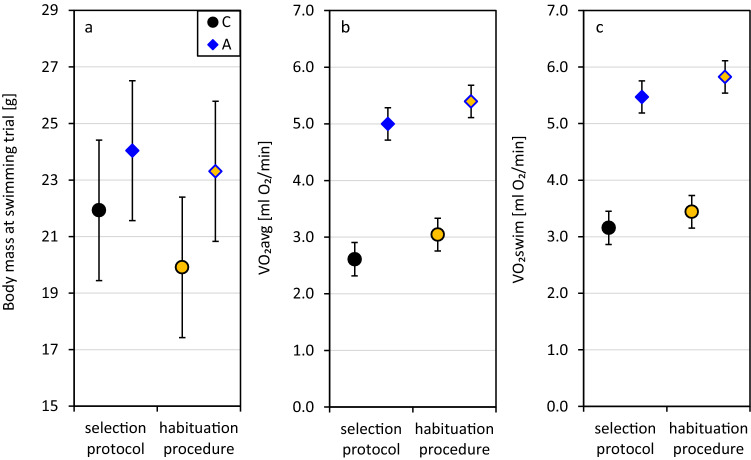
Effects of selection and habituation procedure on body mass and swim-induced rate of oxygen consumption (average: VO_2_avg, and 1-min maximum: VO_2_swim) in bank voles from “control” (C) and “aerobic” (A) lines. The swimming trial was performed on 1026 animals as a part of a selection procedure, and on 100 animals at the conclusion of a three-week habituation procedure. Adjusted least squares means (LSM) and 95% confidence half-intervals (CI) for the metabolic rates were calculated for average (22.4 g) body mass.

Before the experiment, the animals underwent a three-week habituation procedure intended to accustom them to the daily weighing and feces collection. The procedure ended with a swimming trial, performed in parallel to non-habituated animals undergoing standard selection protocol (Table 1). Body mass of 1126 animals that completed the swimming trial in generation 25 of the selection experiment (1026 as a part of regular selection program, and 100 that underwent the three-week habituation) ranged from 13.1 to 37.2 g (mean ± SD: 23.3 ± 3.9 g, Supplementary Table S1, Fig. S1a,b), and did not differ significantly between the A and C lines (*p* = 0.10). Males were heavier than females (*p* = 0.0002, Table 2), and body mass was lower in animals from larger litters (*p* < 0.0001). During the habituation period body mass of the C-line voles gradually decreased over the first two weeks and stabilized in the third week of manipulations, whereas in the A-line voles body mass was stable in the first week and gradually increased in the following two weeks (Supplementary Fig. S2). Consequently, the final body mass was lower than in voles not subjected to the procedure, although the difference was mild and significant only in the C lines (C: 9% difference, *p* < 0.0001; A: 3% difference, *p* = 0.093; selection × habituation interaction: *p* = 0.018, Fig. 2a). The effects of the remaining elements of the model (see Methods) were not significant (p ≥ 0.10).

**Table 2:** Results of ANCOVA models performed on data from all animals which underwent a swimming trial in generation 25, either as a part of selection protocol or following a habituation procedure for the chronic stress experiment.

Variable (abbreviation)	selection direction	sex	habituation procedure	selection × sex	selection × habituation procedure	body mass	selection × body mass	time at swimming	litter number	litter size	respirometry system
**Body mass at swimming trial**											
F	3.71	56.88	15.84	0.38	5.60	n.a	n.a	2.67	2.17	28.55	n.a
df	1,6.26	1,6.46	1,30.2	1,6.46	1,1023	n.a	n.a	1,909	2,59.2	1,937	n.a
p	0.10	0.0002	0.0004	0.6	0.018	n.a	n.a	0.10	0.12	< 0.0001	n.a
**VO**_**2**_**avg**											
F	6.35	0.08	24.73	4.29	0.08	110.27	15.06	2.81	0.03	0.12	5.65
df	1,50.9	1,1100	1,35.6	1,1100	1,1069	1,52.8	1,50.1	1,948	2,69.8	1,656	1,1067
p	0.015	0.8	< 0.0001	0.039	0.8	< 0.0001	0.0003	0.094	1.0	0.7	0.018
**VO**_**2**_**swim**											
F	9.34	0.81	21.22	2.31	0.40	193.66	18.92	1.43	0.23	0.03	2.36
df	1,59.5	1,1103	1,34	1,1101	1,1072	1,58.3	1,55.5	1,943	2,65.5	1,668	1,1068
p	0.003	0.4	< 0.0001	0.13	0.5	< 0.0001	< 0.0001	0.2	0.8	0.9	0.12

Significance of fixed factors, covariates and interactions on body mass measured at the trial, and measures of swimming metabolism: average oxygen consumption (VO_2_avg) and maximum 1-min oxygen consumption (VO_2_swim). n.a. – non-applicable (factor absent from the model).

In the C lines, the average rate of oxygen consumption during the 18-min swimming trial (VO_2_avg) was on average 16.3% lower than the 1-min maximum (VO_2_swim) (mean ± SD: VO_2_avg: 2.66 ± 0.61 ml O_2_/min; VO_2_swim: 3.17 ± 0.52 ml O_2_/min, Supplementary Table S1, Fig. S1). In the A lines, VO_2_avg and VO_2_swim differed on average by 9.0% (VO_2_avg: 5.15 ± 0.82 ml O_2_/min; VO_2_swim: 5.64 ± 0.76 ml O_2_/min). Both VO_2_avg and VO_2_swim increased with body mass, and the increase was steeper in the A than in C lines (selection × body mass interaction: *p* ≤ 0.0003, Table 2). Mass-adjusted VO_2_swim and VO_2_avg were significantly higher in the A than in C lines in the entire range of body masses (*p* ≤ 0.0001). For VO_2_avg, there was a significant interaction between selection and sex: A-line males had significantly higher VO_2_avg than females (*p* = 0.043), an effect non-significant in the C lines (*p* = 0.19; selection × sex interaction: *p* = 0.039). The litter number, litter size, or time of day at the swimming trial did not significantly affect the maximum or average rate of oxygen consumption (*p* ≥ 0.094), but VO_2_avg differed between the two respirometric systems used to perform the measurements (*p* = 0.018) whereas no such difference was observed in VO_2_swim (*p* = 0.12). Animals undergoing the habituation procedure had 11% higher VO_2_avg and 7% higher VO_2_swim than those non-handled (*p* < 0.0001, Fig. 2b,c). The selection × habituation interaction was not significant (*p* ≤ 0.5).

### Analyses set 2: pre-treatment (initial) trait values

The analyses performed for 93 animals that completed the experiment revealed that, despite random assignment, some characteristics differed between the CMS and comfort groups already before the CMS treatment was applied.

**Figure 3:**
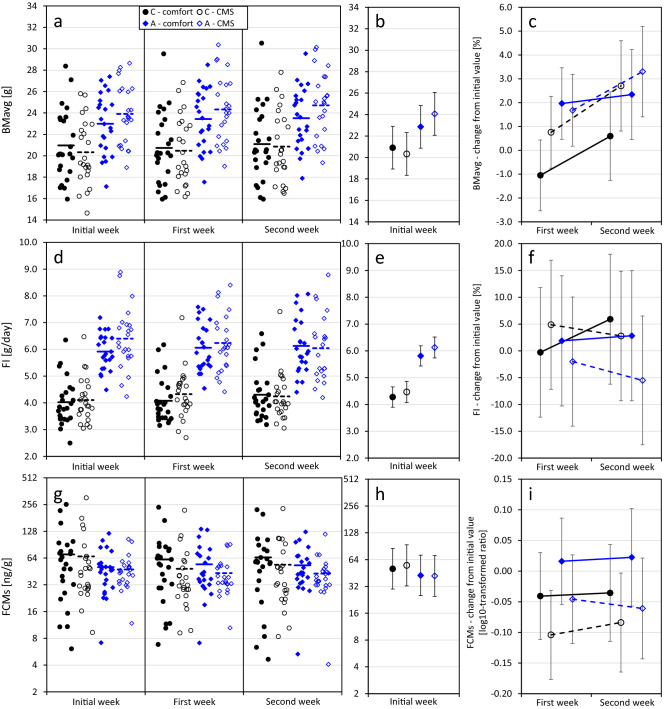
The effects of selection and chronic mild stress (CMS) on bank vole body mass (BMavg), food intake rate (FI), and fecal corticosterone metabolites level (FCMs) measured before (Initial week) and during (First and Second week) the experiment. The experiment was performed on 48 C-line (“control”) and 46 A-line (“aerobic”) voles. Left column – raw data with group means, middle and right columns—least squares means ± 95% confidence intervals, for FI and FCMs calculated for an average (22.4 g) body mass. Results of the experimental treatment (right column) for BMavg and FI are represented by % change, and for FCMs as the log_10_-transformed ratio, relative to the initial value.

Body mass (BMavg), averaged across four days preceding the treatment, ranged from 14.6 to 28.6 g among individuals (mean ± SD: C-lines: 20.65 ± 3.16 g, A-lines: 23.45 ± 2.84 g, Supplementary Table S2, Fig. 3a). Males were heavier than females (*p* = 0.0012, Table 3). An imbalance was found among animals from the two selection directions assigned to two CMS treatment groups: A-line voles assigned to the CMS group tended to be heavier than those assigned to the comfort group (*p* = 0.057), and the A-line voles were heavier than C-line ones in the CMS group, but not in the comfort group (effect of selection tested within groups: CMS: p = 0.016, comfort: *p* = 0.15, selection × treatment interaction: *p* = 0.046, Fig. 3b).

**Table 3:** Results of ANCOVA models performed on initial trait values, measured in animals involved in the CMS-treatment experiment, before the treatment was applied.

Variable (abbreviation)	selection direction	sex	treatment	selection × sex	selection × treatment	body mass	respirometry system	time at swimming
**BMavg**								
F	6.25	33.33	0.51	0.10	4.13	n.a	n.a	n.a
df	1,6.05	1,6.02	1,76	1,6.02	1,76	n.a	n.a	n.a
p	0.046	0.001	0.5	0.8	0.046	n.a	n.a	n.a
**FI**								
F	68.77	0.19	2.20	1.18	0.14	28.59	n.a	n.a
df	1,15.2	1,85.9	1,11.2	1,76	1,11.5	1,69	n.a	n.a
p	< 0.0001	0.7	0.17	0.3	0.7	< 0.0001	n.a	n.a
**FCMs**								
F	0.52	0.15	0.09	0.67	0.15	1.88	n.a	n.a
df	1,6.94	1,9.46	1,75.8	1,5.92	1,76.5	1,85.2	n.a	n.a
p	0.5	0.7	0.8	0.4	0.7	0.17	n.a	n.a
**VO**_**2**_**avg**								
F	120.93	0.03	4.39	1.89	8.83	32.66	1.85	1.06
df	1,7.06	1,76.5	1,5.8	1,69.6	1,6.17	1,78.3	1,73.2	1,75.7
p	< 0.0001	0.9	0.083	0.17	0.024	< 0.0001	0.18	0.3
**VO**_2_**swim**								
F	174.76	0.04	5.83	0.22	10.38	36.73	2.00	2.61
df	1,7.22	1,78.6	1,6.08	1,71.7	1,6.58	1,79.2	1,76.5	1,76.6
p	< 0.0001	0.8	0.052	0.6	0.016	< 0.0001	0.16	0.11

Significance of fixed factors, covariates and interactions on body mass averaged over 4 days (BMavg), apparent daily food intake rate (FI), fecal corticosterone metabolites (FCMs), and measures of swimming metabolism: average oxygen consumption (VO_2_avg) and maximum 1-min oxygen consumption (VO_2_swim). Treatment and its interaction with selection represent differences between the CMS and comfort group resulting from chance effects in group assignment, not the CMS treatment per se. n.a. – non-applicable (factor absent from the model).

Apparent food intake rate (FI), averaged across 6 days preceding the treatment, ranged from 2.5 to 8.9 g/day, and was higher in heavier animals (effect of BMavg: *p* < 0.0001, Table 3, Supplementary Table S2, Fig. 3d). Mass-adjusted FI did not differ between sexes (*p* = 0.7), but was significantly higher in the A than in C lines (A lines: 6.16 ± 0.97 g/day, C lines: 4.06 ± 0.85 g/day, *p* < 0.0001). The FI did not differ between animals assigned to the two CMS treatment groups (*p* = 0.16, Fig. 3e).

Fecal corticosterone metabolite (FCM) levels, averaged across two or four days preceding the treatment, ranged from 6.1 to 305.2 ng/g (C-lines: 68.7 ± 64.6 ng/g, A-lines: 49.1 ± 24.0 ng/g, Supplementary Table S2, Fig. 3g). There were no significant differences between selection directions, sexes or animals assigned to the two CMS treatment groups (Table 3, Fig. 3h).

**Figure 4:**
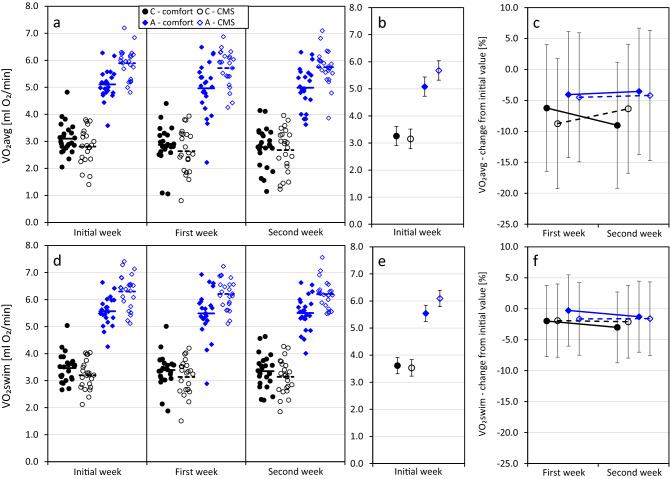
The effects of selection and chronic mild stress (CMS) on bank vole average and maximum swim-induced rate of oxygen consumption (VO_2_avg and VO_2_swim) measured before (Initial week), during (First week), and after (Second week) the experiment. The experiment was performed on 48 C-line (“control”) and 46 A-line (“aerobic”) voles. Left column – raw data with group means, middle and right columns—least squares means ± 95% confidence intervals of trait values calculated for an average (22.4 g) body mass. Results of the experimental treatment (right column) are represented as % change relative to the initial-week values.

The values of VO_2_avg, measured at the onset of the treatment, ranged from 1.40 to 7.20 ml O_2_/min (C lines: 2.96 ± 0.63 ml O_2_/min, A lines: 5.50 ± 0.69 ml O_2_/min) and VO_2_swim ranged from 2.11 to 7.41 ml O_2_/min (C lines: 3.34 ± 0.54 ml O_2_/min, A lines: 5.93 ± 0.69 ml O_2_/min, Supplementary Table S2, Fig. 4a,d). Both of the rates increased with body mass (*p* < 0.0001), but did not differ between sexes (*p* ≥ 0.8; Table 3). In both treatment groups the mass-adjusted VO_2_avg and VO_2_swim were higher in the A than in C lines (*p* < 0.0001). A-line voles assigned to the CMS group had a higher VO_2_avg (*p* = 0.012) and VO_2_swim (*p* = 0.063) than those assigned to the comfort group, but there was no such difference in C lines (*p* ≥ 0.5; selection × group interaction: *p* ≤ 0.024, Fig. 4b,e).

### Analyses set 3: The effects of chronic mild stress (CMS) treatment

Because some characteristics differed between the CMS groups already before the treatment was applied, the effects of the CMS treatment and other results from this set of analyses are reported as either percent change or ratio between in-experiment and the initial values, measured before the CMS *vs* comfort treatment was applied.

The percent change of BMavg from its initial value ranged from -11.3 to 13.7% and tended to be higher in the A lines (mean ± SD: C lines: 0.7 ± 4.6%, A lines: 2.3 ± 3.0%; *p* = 0.070, Fig. 3c, Table 4), but did not differ between sexes (*p* = 0.97). The values were higher in the second than in the first week of the treatment (*p* < 0.0001). CMS treatment did not affect body mass changes (*p* = 0.13), and the effect of selection × treatment interaction was not significant (*p* = 0.3).

**Table 4:** Results of ANCOVA models performed on scopes of deviation from initial trait values during the experiment. Treatment and its interaction with other factors represent the effect of the CMS treatment, relative to the “comfort” group.

Variable (abbreviation)	selection direction	sex	treatment	week	selection × sex	selection × treatment	selection × week	treatment × week	sex × treatment	selection × treatment × week	body mass	respirometry system	time at swimming
**BMavg**													
F	4.18	0.00	2.35	26.46	0.08	1.17	2.16	2.03	n.a	0.75	n.a	n.a	n.a
df	1,9.32	1,82.4	1,79	1,89	1,81.7	1,79	1,89	1,89	n.a	1,89	n.a	n.a	n.a
p	0.070	1.0	0.13	< 0.0001	0.8	0.3	0.15	0.16	n.a	0.4	n.a	n.a	n.a
**FI**													
F	2.56	1.83	1.85	0.17	0.65	3.58	3.09	11.34	n.a	1.02	0.36	n.a	n.a
df	1,7.49	1,80.8	1,6.07	1,88.4	1,71.3	1,6.22	1,86.9	1,86.9	n.a	1,86.8	1,73.9	n.a	n.a
p	0.15	0.18	0.2	0.7	0.4	0.11	0.082	0.001	n.a	0.3	0.5	n.a	n.a
**FCMs**													
F	2.11	0.24	5.61	0.06	0.67	0.10	0.23	0.01	n.a	0.39	0.09	n.a	n.a
df	1,16.4	1,16.1	1,75.2	1,7.1	1,10.2	1,75.5	1,7.02	1,80.2	n.a	1,80.2	1,62.7	n.a	n.a
p	0.17	0.6	0.020	0.8	0.4	0.8	0.6	0.9	n.a	0.5	0.8	n.a	n.a
**VO**_**2**_**avg**													
F	0.48	0.65	0.01	0.00	0.16	0.01	0.04	0.63	5.76	0.74	3.18	0.00	0.03
df	1,7.3	1,10.1	1,71.9	1,3.67	1,5.23	1,73.4	1,82.9	1,82.8	1,71.8	1,82.8	1,88.7	1,136	1,156
p	0.5	0.4	0.9	1.0	0.7	0.9	0.8	0.4	0.019	0.4	0.078	1.0	0.9
**VO**_**2**_**swim**													
F	0.15	0.08	0.01	0.06	0.00	0.19	0.00	0.23	n.a	0.00	0.27	0.42	0.42
df	1,7.55	1,9.05	1,70.4	1,1.99	1,5.49	1,73.3	1,81.9	1,81.8	n.a	1,82.2	1,83.1	1,140	1,160
p	0.7	0.8	0.9	0.8	1.0	0.7	0.9	0.6	n.a	1.0	0.6	0.5	0.5

Significance of fixed factors, covariates and interactions on body mass averaged over 4 days (BMavg), apparent daily food intake rate (FI), fecal corticosterone metabolites (FCMs), and measures of swimming metabolism: average oxygen consumption (VO_2_avg) and maximum 1-min oxygen consumption (VO_2_swim). n.a. – non-applicable (factor absent from the model).

The scope of change of further traits was not affected significantly by body mass (*p* ≥ 0.5, except for VO_2_avg, for which a positive trend appeared, *p* = 0.078), and did not differ systematically between sexes (*p* ≥ 0.18) or the two treatment weeks (*p* ≥ 0.7). The interactions present in the models but not mentioned in the text below were not significant (*p* ≥ 0.2). Within all traits the repeated measures factor of individual was significant (*p* ≤ 0.001, Supplementary Table S3). Except for FCMs, in-experiment changes in all traits varied among replicate lines (*p* ≤ 0.058). The changes in FI varied among experimental blocks (*p* < 0.0001).

The percent change of FI from its initial value ranged from -25.8 to 46.3% (C lines: 5.9 ± 12.7%, A lines: -0.5 ± 9.4%). Between the first and the second treatment week the values increased in the C lines and decreased in the A lines, resulting in a considerable interaction (*p* = 0.08) and nearly significant difference between the lines at the end of the treatment (first week: *p* = 0.4; second week: *p* = 0.06, Fig. 3f, Table 4). The pattern of FI changes throughout the treatment differed also between treatment groups (treatment × week interaction: *p* = 0.001): in the comfort group FI increased between the first and second week (effect of week: *p* = 0.009), whereas in the CMS group it decreased (*p* = 0.041), resulting in a significant difference between the groups in the second week (first week: *p* = 0.8, second week: *p* = 0.02). The selection × treatment interaction was not significant (*p* = 0.11).

The log_10_-transformed ratio of FCMs measured during the treatment to its initial value ranged from -0.60 to 0.29 (C lines: -0.07 ± 0.15, A lines: -0.02 ± 0.16) and did not differ between selection directions (*p* = 0.16, Fig. 3i, Table 4). The ratio was lower in the CMS than in the comfort group (*p* = 0.020), i.e., the fecal corticosterone metabolites decreased in response to the CMS treatment, with no significant selection × treatment interaction (*p* = 0.8).

The percent change of VO_2_avg from its initial value ranged from 66.6 to 87.8% (C lines: -6.1 ± 24.6%, A lines: -2.4 ± 12.3%). The CMS treatment affected males and females differently (interaction: p = 0.019): in males the VO_2_avg tended to decrease more in the CMS than in the comfort group (p = 0.077), whereas in females the effect of treatment was not significant (*p* = 0.11, Table 4). The scope of change did not differ between selection directions (*p* = 0.5) and was not affected by selection × treatment interaction (*p* = 0.9, Fig. 4c).

The percent change of VO_2_swim from its initial value ranged from -47.0 to 76.2% (C lines: -1.8 ± 15.8%, A lines: -1.2 ± 8.6%). Selection and CMS treatment did not significantly affect the scope of change (*p* ≥ 0.7) and the selection × treatment interaction was also non-significant (*p* = 0.7, Table 4, Fig. 4f).

## Discussion

This study confirmed that the artificial selection for high swim-induced 1-min maximum aerobic exercise metabolism (VO_2_swim) in bank voles has resulted not only in an immense increase in the directly selected trait, but also in the metabolic rate averaged over the entire swimming trial (VO_2_avg). In generation 25, voles from the selected (A) lines achieved 80% higher VO_2_swim and 99% higher VO_2_avg than those from the unselected control (C) lines (Fig. 2b,c). Although the current study did not involve behavioral observations, the more pronounced increase in VO_2_avg than in VO_2_swim suggests an increase in not only peak swimming effort but also in proportion of time spent on active swimming. This is in agreement with results of direct observations of Jaromin et al.^38^, who reported an increase in time spent on active swimming rather than floating in the A lines. The A-line voles also tend to be larger than C-line ones, and have substantially higher basal metabolic rate^35^ fueled by increased food intake^43^. Chronic stress is known to reduce mobility in a swimming trial and disrupt food intake and body mass^33^, therefore it was particularly interesting to check if selection in the A lines has affected sensitivity to chronic stress reflected in changes observed in these traits. Finally, because of the central role of glucocorticoid hormones in mediating the stress response^3,7,20,21^, we measured corticosterone non-invasively, via its metabolites in the feces^44^.

In bank voles, weighing and sampling fresh feces require handling and temporarily placing the animals in empty containers. Although the procedure is brief (20 min), it is stressful, particularly for animals with infrequent prior contact with humans^45^. However, animals are able to habituate and reduce the stress response to a known stressor, particularly if it appears repeatedly and the animal can predict its occurrence^20,45–47^. Hence, it is commonly advised to habituate animals to experimental procedures through repeated exposure, an approach expected to reduce the bias caused by novelty of unintentional stressor^47–49^. Here, we preceded the proper experiment with three weeks of daily weighing and feces collection sessions. The habituation procedure concluded with a swimming trial, performed in parallel with naïve animals from the base colony. This gave us an opportunity to explore the rarely-reported effects of such a habituation procedure.

After three weeks of habituation, body mass of the voles was lower than in those not subject to the habituation procedure, although difference was significant only in the C lines (Table 2, Fig. 2a, Supplementary Figs S1, S2). A reduction of body mass or inhibition of growth are among common effects of anxiety and chronic stress^2,3,50^. Therefore, it could be argued that behavioral habituation to the experimental procedures has not been achieved, and that the animals continued to perceive the daily manipulations as stressful. However, in the A lines the body mass growth was halted only during the first few days, and in the C lines the body mass stabilized in week three of the habituation period (Supplementary Figure S2). Thus, the pattern of body mass changes indicates that the voles have eventually habituated to the daily manipulations, although the process took much more time in C-line voles. Moreover, chronic stress typically results in decreased activity in a swimming trial (^33^, but see^51,52^), whereas in our experiment the swimming performance, measured as either the maximum or average aerobic metabolic rate during the swimming trial, increased in voles from the habituated group, both in the C and A lines (Table 2, Fig. 2b,c, Supplementary Fig. S1). Similarly, in mice undergoing daily restraint stress, body mass gain was halted only temporarily, and after two weeks of treatment the animals had an increased activity in a swimming trial and reduced glucocorticoid response to the restraint stress, leading to the observations being interpreted as signs of habituation^9^. In rodents, even a brief handling session disrupts the housing stability and agitates an animal for far longer than the session lasts^45,53^. Thus, both the decreased body mass and the increased aerobic exercise performance in the habituated group could be an effect of the additional daily locomotor activity. Normally, voles from the C lines have a lower home-cage activity than those from A lines^54^, so it is understandable that the additional activity could have a more profound effect on their energy balance, and hence their body mass. However, in the case of animals whose locomotor activity under laboratory maintenance is certainly lower than in nature, an increased activity and mildly reduced body mass (ca 9% in C lines, and 3% in A lines, compared to the animals not subjected to the procedure) can be considered as a healthy outcome, rather than a chronic-stress response. The increased swimming performance in voles from the habituated group could be also explained by a purely behavioral mechanism. Although the daily manipulations had not much in common with the swimming procedure, except the handling itself, the animals might have become accustomed to the possibility of being disturbed, and the reduction of the stress response to such disturbance allowed them to shorten the delay in mounting the proactive response – and hence they performed better in the test. Therefore, both the mild reduction of body mass and an increase in swimming performance could be attributed to positive effects of the habituation.

The most pronounced effect of the chronic mild stress (CMS) procedure was a decrease in the fecal corticosterone metabolites level (Fig. 3g,i, Table 4). Levels of metabolites did not differ between the C and A lines at the onset of the experiment (Fig. 3h), in line with our earlier findings that baseline plasma corticosterone level did not differ between the two line types^28,40^. After applying the CMS procedure FCMs level decreased by about 15%, but similarly in both line types (Fig. 3i). Thus, the CMS procedure gave a result opposite to that commonly expected to occur when recurring stressors are chronically but unpredictably stimulating the glucocorticoid release^2,3,7^. Moreover, the voles’ health and functioning was apparently not compromised. These observations may suggest that the voles were resistant to CMS, or even that such conditions were less stressful than the standard, monotonous housing. However, although in the majority of studies chronic stress resulted in elevation of integrated glucocorticoid levels, no effect or a reduction were also common^2^. In particular, a reduction of glucocorticoids levels has been observed in several strains of mice exposed to chronic stress^30^, in horses experiencing compromised welfare^55^ and in humans suffering from a particular form of depression^3^. Additionally, conclusions based on fecal corticosterone metabolite levels should be treated with caution, both because these values are not a straightforward representation of the plasma level of metabolically active hormones^56^, and because the strength of the hormone’s signaling can be modified by changes in expression of its receptors^57^. Although chronic stress can affect the receptor expression^58,59^, we could not test if a similar change occurred in the voles. Therefore, we still cannot exclude the possibility that the voles undergoing the CMS procedure were actually chronically stressed.

During the proper experiment the mass of C-line voles did not change significantly, while it continued to increase in the A-line ones (Fig. 3a,c, Supplementary Fig. S2). Body mass was not affected by the CMS treatment, despite the slight reduction of food intake in the CMS group at the second week of the treatment (Fig. 3f). The methodology applied in the experiment did not allow to investigate the possible metabolic or behavioral sources of the discrepancy. Although little is known on how changes in the amount of matter passing through the digestive tract affects concentration of glucocorticoid metabolites measured in feces, we considered the possibility of diluting effect of increased food intake^60^. Instead, the observed reduction of food intake in the CMS group strengthens the conclusion that the treatment reduced fecal corticosterone metabolite level in voles.

Based on a number of studies in which chronic stress was used to induce depression-like state in rodents^33,50^, we expected the CMS procedure to reduce activity of voles in a swimming trial. Surprisingly, we observed the effects of CMS only in males, for which the average swim-induced rate of oxygen metabolism decreased to a greater extent in the CMS group (10%) than in the comfort group (4%), whereas no such effect was observed in females. The finding is in line with the concept that the behavioral and physiological responses to stress differ between sexes^61^. In particular, chronic stress reduced swimming activity in the first swimming trial in male, but not in female rats^62^. In voles, the CMS procedure reduced the males’ average, but not the maximum rate of swim-induced oxygen consumption (Table 4), suggesting that the exposure to stressors affected only the inclination towards an enduring activity, but not the scope of burst performance. Although we expected the A-line voles to be more susceptible to CMS, the effect of CMS on swimming performance did not differ between the C and A lines. Arguably, the distinction could be reduced by excluding from the experiment the animals that were not able to complete the swimming trial because of drowning or repeated diving, but as only seven individuals were excluded, the bias could not have been severe. Therefore, none of the traits we measured provided evidence that selection had affected the voles’ sensitivity to chronic stress.

In conclusion, we did not find support for the hypothesis that evolution of increased aerobic exercise performance under an acute challenge is traded off by an increased vulnerability to chronic stress. However, the chronic stress protocol we applied had relatively little effect on the traits we measured, or the response was opposite to what we expected. These counter-intuitive results may indicate that the animals were not particularly sensitive to chronic stress, or that the group not subjected to the chronic stress paradigm was not free of chronic stress, either. Interestingly, the habituation procedure consisting of presumably mildly stressful daily events resulted in increased swimming performance, and the CMS conditions reduced fecal corticosterone metabolites. Thus, it could even be speculated that recurrent mild stressors, disrupting stable but monotonous life, can be beneficial for animals. Such a conjecture, however, would require confirmation in experiments explicitly designed for that purpose, before it could be proposed as a basis for recommendation in animal welfare practices.

## Materials and methods

### Animal model and husbandry

This work was performed on bank voles (*Myodes* = *Clethrionomys glareolus* Schreber 1780) from generation 25 of an ongoing artificial selection experiment maintained at the Jagiellonian University (Poland). The rationale, history and protocols of the experiment have been presented in our earlier papers^28,34,35,39,40^. Briefly, the colony was established based on about 320 wild voles captured in 2000 and 2001. After 5–6 generations of random breeding, the selection experiment has been started. In the selected, “aerobic” (A) lines the selection criterion is the maximum 1-min rate of oxygen consumption (VO_2_swim), achieved during a swimming trial. The VO_2_swim values used as selection criteria are mass-adjusted (residuals from ANCOVA including also other covariates and cofactors). Four replicate lines for both selected and unselected control (C) lines are maintained (to allow valid tests of the effects of selection; Henderson, 1997), with 15–20 reproducing families in each of the 8 lines (which avoids excessive inbreeding). Since generation 18, VO_2_swim of the A lines exceeds that of the C lines by more than 60% (Supplementary Fig. S1b).

The animals were maintained in standard, polypropylene mouse cages with sawdust bedding, at constant temperature (20 ± 1 °C) and photoperiod (16:8 light:dark; light phase starting at 2am). At the age of 17 days the animals were weaned, marked temporarily by fur clipping and kept in family groups. At the age of about 34 days, all individuals were marked permanently with mouse ear tags (model 10,005–1; National Band and Tag, Newport, KY; mass 0.18 g) and later maintained in same-sex groups of three individuals in model 1264C cages or up to four individuals in a larger model 1290D cages (Tecniplast, Bugugiatte, Italy). The thick layer of sawdust allowed the animals to burrow under its surface, and it contained fragments large enough for the animals to gnaw on. Cages were changed every 5–14 days, depending on the number of animals in the cage, size of the cage and their cleanliness. Water and food (a standard rodent chow: 24% protein, 4% fat, 4% fiber; Labofeed H, Kcynia, Poland) were provided ad libitum. Every day all cages were visually inspected for presence of food and water or dead animals. The colony was under supervision of a qualified veterinary surgeon. During any kind of measurements, if symptoms of poor condition were observed in an animal (problems with breathing or moving, injury, etc.), it was removed from the experiment. Depending on judgment of the observer or animal care personnel, it was either allowed to recover or euthanized.

All the breeding, selection and experimental procedures were approved by the Local Ethical Committees in Krakow, Poland (decision no. 170/2014 – 1^st^ Local Ethical Committee for Animal Experiments, Faculty of Pharmacy, Jagiellonian University Medical College in Kraków; 257/2017 – 2^nd^ Local Institutional Animal Care and Use Committee, Institute of Pharmacology Polish Academy of Sciences in Kraków). In generation 25, the standard selection test was completed in 1026 individuals (849 from the A lines and 177 from the C lines), including all available A-line animals and typically one male and one female from each C-line family.

### Selection test: swimming trial

The selection VO_2_swim test was performed at the age of 75–85 days, between 8:00 and 18:30 h, as described in our earlier reports^28,34,37^. The setup, equipment and calculations were identical to these described in^28^. At the day of measurement, home cages were transferred from the housing room to the laboratory. An animal was removed from its cage shortly before the start of its measurement, weighed, and placed in a temporary container where it stayed for several minutes until the test started. The trial was performed in 15 cm diameter 3-L glass jars (respirometric chambers) partly filled with water. To ensure that the metabolic response was related to physical activity rather than thermoregulatory burden, and to avoid a possibly confounding effect of reaction to cold stress, the water temperature was set at 38 °C. The animal was gently placed on the surface of water and allowed to swim freely. The test lasted for up to 18 min, unless an individual began to drown or oxygen consumption rapidly decreased. After the swimming trial the animal was wiped with paper towel, returned to its home cage fitted with fresh bedding, and placed under a heating lamp which supported fur drying. The cages were then returned to the colony.

### Experimental design overview

The experiment comprised two phases: three-week habituation, in which all animals were treated in the same way (days D-21 to D0), and two-week experiment proper, in which the CMS procedure was applied to half of the animals (days D+1 to D+14; Table 1; Fig. 1). All the work was performed in three nearly-balanced blocks, starting in 15-day intervals.

During the habituation period the animals could get accustomed to the daily weighing and fecal collection sessions that continued throughout the proper experiment. The habituation was performed on 49 A-line 69 C-line animals, sampled approximately equally from each of the replicate lines, one individual from a full-sib family. The animals were 54–64 days old at the beginning of this period. Every day the animals were handled, weighed and moved for 20 min to an empty cage, which enabled collection of freshly deposited feces. The procedures were suspended only on days D0, D+7 and D+14, when VO_2_swim was measured. The swimming trials followed the same procedure as in the selection test. After the D0 trial, 46 A-line and 48 C-line voles were sampled from those that completed the trial without incidents of drowning or diving attempts. These animals were assigned to two experimental groups, 47 animals each. One of the experimental groups was moved to a separate animal housing room where chronic mild stress (CMS) protocol was applied (Table 1). The second group remained undisturbed aside from the daily weighing and fecal collection that continued for both groups throughout the experiment. Housing in the separate rooms prevented transmission of both the environmental stimuli associated with the CMS treatment and visual, olfactory or acoustic signals generated by the animals. The swimming trials at D+7 and D+14 were performed on animals from both groups, in randomized order. On D-7, D+1, and D + 8 animals received weighed portions of approximately 105 g of standard food, and food remaining in feeder was weighed one day before each swimming trial.

Data were collected in three sets, representing three weeks, each concluded with a swimming trial: the last week of the habituation procedure (initial measures), and the first and the second week of the proper experiment. Five types of data were collected: the maximum and average rate of oxygen consumption during swimming (VO_2_swim and VO_2_avg), apparent food intake rate (FI) calculated from mass of food removed from feeder over 5 or 6 days preceding a swimming trial, and body mass (BMavg) and fecal corticosterone metabolites level (FCMs)^44^, both representing four days preceding a swimming trial.

### Habituation procedure

At day D-22 the animals were weighed and separated into model 1264C cages (Tecniplast, Bugugiatte, Italy). Unlike rats or mice, bank voles are solitary in nature^64^, and thus social isolation is unlikely to elicit stress-related disorders. Aside from being housed individually, the housing conditions were identical to that applied in the colony. Daily habituation sessions started at D-21. In mice, 14 days of daily handling was sufficient for habituating the animals to human presence^45^. The additional seven days we applied allowed collecting the initial data unbiased by the novelty of the daily routine and the experimental procedures applied afterwards.

Between 8:00 and 11:00am, each animal was removed from its home cage by neck scruff or in cupped hand, weighed in an opaque plastic cup (approximately 18 cm high and 8 cm in diameter) and placed in an empty, “sampling” cage of the same model as the housing cage, with no sawdust or access to food or water. After 20 min the animal was returned to its home cage. Feces deposited in the empty cage were collected starting from D-2 for the first block and D-7 for the second and third blocks.

### Experimental groups establishment

During the habituation period two A-line animals died and three C-line ones were recognized as diabetic and excluded from the experiment (diabetes appears in a few percent of bank voles both in laboratory conditions and wild populations^65^). The swimming trial performed at the end of the habituation (D0) was interrupted for four C-line animals which started to drown; another two C-line and one A-line animal were repeatedly diving which biased the measurement of their oxygen consumption. These seven animals were excluded from the experiment. The experimental blocks were balanced by excluding six excessive C-line animals, randomly chosen from over-abundant combinations of sex and replicate line. Thus, the proper experiment was performed on 48 C-line and 46 A-line voles (94 animals in total).

The animals were assigned to two treatment groups (comfort and CMS), 47 animals each. The assignment ensured that the number of animals of either sex from each replicate line was balanced between the groups. Within the replicate line × sex subgroup the assignment to the treatment groups was based on randomization blind with respect to all known or measured characters, particularly the age, mass or swimming performance.

### Chronic mild stress (CMS) treatment

The daily manipulations introduced in the habituation procedure were continued on animals from both experimental groups throughout the experiment, and the order in which it was applied to the two groups within a given day was randomized. Animals from the CMS group were subjected to a series of mild stressors listed in Table 1. The procedure was adapted from protocols inducing the state of chronic stress in mice or rats^66–68^. Two types of stressors were applied during each day. The series was arranged in a way that allowed the stressors applied during nighttime (18:00 to 2:00) and dawn (2:00 to 8:00) to be automated (disrupted light regime, intermittent noise or vibration), and those requiring manual operations were applied during daytime (8:00 to 18:00). Immediately after the daily handling, weighing and fecal collection the daytime stressor was initiated by either placing the animal in a new cage, containing no bedding or with a bedding thoroughly soaked with lukewarm water, or returned to the home cage but without access to water or food. After 8 h the animals were returned to their home cages or access to food and water was restored, respectively to the type of stressor applied.

One CMS-group animal died in the first week of the experiment, and data collected from this individual were not used in the statistical analyses.

### Feces collection and corticosterone metabolite analysis

Immediately after the animals were returned to their home cages, feces deposited in the “sampling” cages were collected using tweezers into empty Eppendorf tubes (0.5 ml, one tube per individual per session). Feces contaminated with urine were discarded, as bank vole urine contains varying amounts of corticosterone metabolites^69^. The samples were stored on ice for up to 2 h until frozen in -20 °C.

After completion of all experimental procedures, the samples were dried overnight at 80 °C. Because we observed a tendency for body mass to drop after the swimming trial, a decrease usually recovered over the following day or two, we assumed the fecal samples collected during the post-swimming recovery period could be biased by changes in the animal’s metabolism and food intake. Therefore, we excluded the samples collected during the two days following each swimming trial. The remaining samples were used to create three pools representing the four days preceding each of the three swimming trials (days D-4 or D-2 to D-1, D+3 to D+6 and D+9 to D+13). The pooled samples were weighed, ground in a mortar, and a 50 mg subsample was placed in 1.5 ml Eppendorf tubes with 1 ml 80% methanol (POCh, Poland). The tubes were shaken for 30 min, then centrifuged for 10 min at 2500 g. A 0.5 ml aliquot of the supernatant was transferred to a separate Eppendorf tube and stored in -20 °C. The trios of samples taken from an individual vole were extracted within the same batch, and the order in which these trios were extracted was randomized within blocks.

Fecal corticosterone metabolites (FCMs) in the extracts were measured using a 5α-pregnane-3β,11β,21-triol-20-one enzyme immunoassay, which measures metabolites with a 5α-3β,11β-diol structure. For a more detailed description of the used methods and the assay (e.g. antibodies and inter- and intra-assay variance), see^70^. The method was validated and proven suitable for bank voles^69^. In that particular validation experiment, it was shown that the intestinal gut passage time of corticosterone in bank voles is ca. 6–8 h, from which it can be assumed that the samples collected in our experiment represented corticosterone released in the “early morning”, shortly after the onset of the light phase. All three samples of an individual were analyzed in the same batch, but the order of individuals and samples within individual was randomized.

### Statistical analyses

Three sets of analyses were performed to test (1) effects of the habituation procedure, (2) differences between the comfort and CMS groups at the onset of the treatment, and (3) effects of the CMS treatment. The first set of analyses was performed on data from all individuals from generation 25 which completed the VO_2_swim test: 1026 individuals tested as a part of the regular selection program (not included in the habituation procedure) and 100 included in this specific experiment (which passed the habituation procedure). This set contained data on animals’ body mass and rates of oxygen consumption (VO_2_swim, VO_2_avg) during their first swimming trial. The second and the third set of analyses were performed on data from the 93 individuals that completed the whole experiment. This data set contained data from measurements repeated three times per individual, taken during the last few days of the habituation, i.e., just before the experiment proper (“initial”), and the two weeks of experiment.

The analyses were performed with cross-nested mixed ANCOVA models, using SAS v. 9.4 (SAS Institute, Inc., Cary, NC, USA) Mixed procedure (with REML method of estimation and variance components restricted to positive values). All the models included selection direction (A *vs.* C lines) and sex as the main fixed factors, random effect of replicate line (nested within selection direction), and body mass as a covariate (unless body mass was the subject of analysis). The hierarchical structure of the statistical model (replicate lines nested in selection direction) is required to allow a proper distinction of the effects of selection from random genetic effects, such as the genetic drift^63^. Models for the measures of swimming performance included respirometric system as additional fixed cofactor, and time at the start of measurement as an additional covariate. This basic model structure was further expanded to accommodate additional factors adequate for specific analyses:

1. In the first set of analyses the models included a fixed factor determining if the animals was subject to the habituation procedure or not. The models included also a random factor of family (nested in replicate line) to properly handle non-independence of observations obtained on individuals from the same full-sib families, litter number of a particular family as a cofactor (the 1st, 2nd, and 3rd or further litter), and litter size as a covariate. As animals involved in the selection protocol are not weighed daily, the single body mass measured immediately before the swimming trial was used as a covariate.
2. The second analysis was performed for data obtained in the last few days of habituation period (just before applying CMS treatment): 4-day average body mass (BMavg), 6-day average daily food intake rate (FI) and 2- or 4-day average fecal corticosterone metabolites level (FCMs), and for the average and maximum swim-induced metabolic rates (VO_2_avg and VO_2_swim) measured at day D0. The models included a fixed factor of the CMS treatment (CMS or comfort groups), an interaction between treatment and selection direction, a random factor of experimental block, and BMavg as covariate. Diagnostic graphs (histograms of residuals, residual *vs* quantile plots, and residuals *vs* predicted values plots) showed that values of FCMs were right-skewed, and were therefore log_10_-transformed prior to the analyses.
3. The third, most important set of analyses was applied to similar traits as in the second set (BMavg, FI, FCMs), but measured in the final few days of the first and second week of the CMS treatment, and VO_2_swim and VO_2_avg measured on days D+7 and D+14 of the treatment. Unfortunately, despite best efforts put into group randomization, the second set of analyses revealed that the experimental groups (CMS *vs* comfort) varied in terms of swimming performance already in the “initial” week, before the CMS treatment was applied (see Table 3, Figs. 3, 4). Therefore, to correct for the discrepancy, the effect of CMS treatment on BMavg, FI, VO_2_swim and VO_2_avg was tested for values recalculated to represent percent change from the initial values (measured just before applying the treatment). Only for FCMs, which was right-skewed, the deviation was represented by log_10_-transformed ratio of the in-experiment and initial values (which is equal to difference between log_10_-transformed in^-^experiment and initial values of FCMs). The results obtained in the first and second week of the treatment were analyzed within one, repeated measures model. Therefore, in addition to the main effect of CMS treatment, the model included also the week of experiment (first or second) as a repeated measures (within-individual) fixed factor and a random effect of individual. The preliminary models assumed either equal or unequal residual variance (compound symmetry or unstructured covariance structure), and models with lower AIC (Akaike Information Criterion) were chosen for the final models.

In all the above analyses the initial models included interactions among the main fixed categorical factors (selection, sex, handling or experimental treatment and week, where applicable), between the main fixed factors and BMavg, and all the respective random interactions with the replicate line. The models were then step-wise reduced by removing non-significant interactions (*p* > 0.05). However, the interactions between selection direction and sex, and between selection direction, experimental treatment and week of experiment were a priori considered meaningful from the biological and experimental point of view, and were retained in the models irrespective of their significance. In the analyses for metabolic rates in the first set of analyses the interaction between selection direction and body mass was significant, and the final model had to retain heterogeneous slopes. Therefore, the effect of selection was tested for the average (23 g), minimal (16 g) and maximal (32 g) body mass relevant for both selection groups (using ”at” option in SAS “lsmeans” statement), but since it was highly significant in the entire range of body masses, only results adjusted for the average body mass are reported.

In all analyses the Satterthwaite’s approximation was used to calculate the effective degrees of freedom (df) for t tests or the denominator df for F tests (i.e., the df was computed from a combination of the dfs of respective random grouping effects and residual term, weighted by variance contribution of the terms;^71^. Thus, the dfs could take any real value between df of the random factor and df of the residual term. Significance of the random effects was tested with the likelihood ratio (LR) test, based on results from models with the same structure as described above, but with variance components not restricted to positive values (“nobound” option in SAS Mixed procedure).

In set 3, several outliers were recognized and excluded from the analyses. Because values for these analyses were calculated from an initial and one of the two measurements taken during the experiment, either one or both data points representing a particular individual were considered as outliers. The exclusion was applied to two data points from one individual in FI analysis, one data point in FCMs analysis, four data points from two individuals in VO_2_avg analysis and five data points from three individuals in VO_2_swim analysis (see data in left columns of Figs. 3 and 4, and Supplementary data file containing raw data set). The absolute values of studentized residuals of all excluded points were ≥ 2.7, and within excluded individuals the studentized residual of at least one of the points was ≥ 3.0.

### Ethics approval

All the breeding, selection and experimental procedures were approved by the Local Ethical Committees in Krakow, Poland (decision no. 170/2014 – 1^st^ Local Ethical Committee for Animal Experiments, Faculty of Pharmacy, Jagiellonian University Medical College in Kraków; 257/2017 – 2^nd^ Local Institutional Animal Care and Use Committee, Institute of Pharmacology Polish Academy of Sciences in Kraków), acting in accordance with the legislation of Poland (Journal of Laws of the Republic of Poland 2015/266) and European Union (directive 2010/63/EU). This study is reported in accordance with ARRIVE guidelines (https://arriveguidelines.org).

## Supplementary Information

Supplementary Information 1.

Supplementary Information 2.

## Abbreviations

HPA axis: Hypothalamic–pituitary–adrenal axis
A line: Line of bank voles selected for high aerobic exercise metabolism achieved during swimming (“Aerobic”)
C line: Non-selected line of bank voles (“Control”)
BMavg: Body mass (g) averaged from measurements performed daily for four days preceding a swimming trial
FI: Apparent food intake rate (g/day) calculated from mass of food removed from feeder over 5 or 6 days preceding a swimming trial
FCMs: Fecal corticosterone metabolites level (ng/g) in a sample representing four days preceding a swimming trial
VO_2_swim: Maximum 1-min rate of oxygen consumption achieved in a swimming trial (ml O_2_/min)
VO_2_avg: Average rate of oxygen consumption during the swimming trial (ml O_2_/min
