# Supplementary material for: Evolution of an increased performance under acute challenge does not exacerbate vulnerability to chronic stress
Source: Sci Rep. 2022 Feb 8;12:2126. doi: 10.1038/s41598-022-06060-7 (PMC8825808; doi:10.1038/s41598-022-06060-7)
Supplement: Supplementary file 1 — Supplementary Information 1. [file 41598_2022_6060_MOESM1_ESM.docx]

**Evolution of an increased performance under acute challenge does not exacerbate vulnerability to chronic stress**

**Authors**

Małgorzata M. Lipowska^1,^*, Edyta T. Sadowska^1^, Rupert Palme^2^, Paweł Koteja^1^

^1^ Institute of Environmental Sciences, Jagiellonian University, Kraków, Poland

^2^ Department of Biomedical Sciences, University of Veterinary Medicine, Vienna, Austria

* Correspondence: malgorzata.lipowska@doctoral.uj.edu.pl

**Supplementary materials**

**Figures**

**
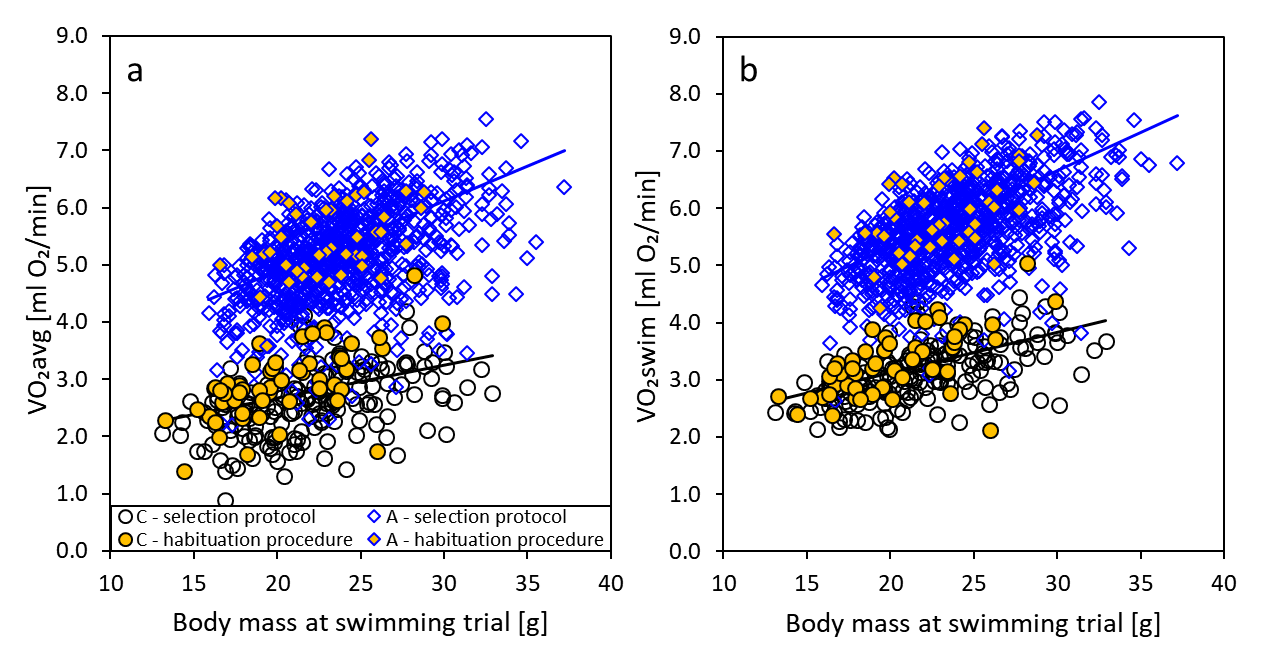
**

**Supplementary Fig. S1** **The relation between the average (a) and maximum (b) swim-induced rate of oxygen consumption (VO_2_avg and VO_2_swim) and body mass in bank voles from “control” (C) and “aerobic” (A) lines, undergoing habituation procedure (yellow symbols, 100 animals) or involved in a selection protocol (empty symbols, 1026 animals).**

**
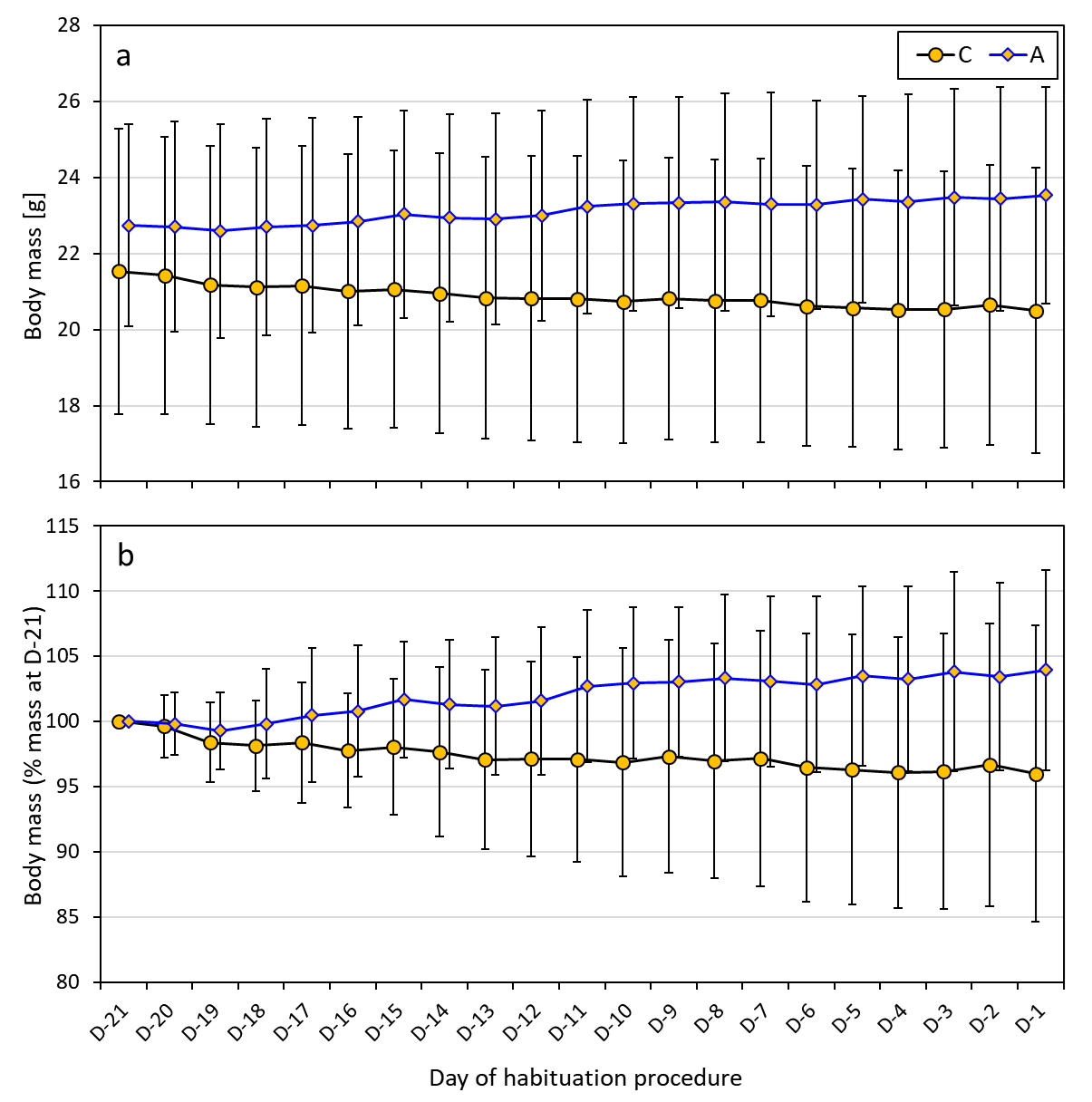
**

**Supplementary Fig. S2 Body mass (mean ± SD) of 54 animals from the “control” (C) and 46 animals from the “aerobic” (A) lines in consecutive days of the habituation procedure.** Top panel – raw values, bottom panel – percent change an individual’s body mass relative to its mass at D-21. The calculation did not involve the 2 animals that died during the procedure, 3 that were recognized as diabetic and 7 in which the swimming trial at D0 was not successful.

**Tables**

**Supplementary Table S1 Sample size and mean ± standard deviation of body mass and swim-induced metabolic rates of all animals that underwent a swimming trial in generation 25.** Animals from two line types (“control” and “aerobic”) were tested either as a part of selection protocol or following a habituation procedure for the chronic stress experiment.

| Variable (abbreviation) | Variable (full name) | “control” (C) lines | |  | “aerobic” (A) lines | |
| --- | --- | --- | --- | --- | --- | --- |
|  |  | selection protocol | habituation procedure |  | selection protocol | habituation procedure |
| N | number of individuals | 177 | 54 |  | 849 | 46 |
|  | body mass at swimming trial [g] | 21.9 ± 4.1 | 20.3 ± 3.6 |  | 23.8 ± 3.7 | 23.1 ± 2.9 |
| VO_2_avg | average oxygen consumption in a swimming trial [ml O_2_/min] | 2.58 ± 0.58 | 2.94 ± 0.63 |  | 5.13 ± 0.82 | 5.50 ± 0.69 |
| VO_2_swim | maximum 1-min oxygen consumption in a swimming trial [ml O_2_/min] | 3.13 ± 0.50 | 3.31 ± 0.56 |  | 5.63 ± 0.76 | 5.93 ± 0.69 |

**Supplementary Table S2 Sample size and mean ± standard deviation of values measured in the animals used in the experiment.** Data collected over three consecutive weeks: initial (last week of the habituation procedure), and first and second week of the experiment, from animals representing two line types (“control” and “aerobic”) and divided into two experimental groups (comfort and CMS – Chronic Mild Stress).

| Variable (abbreviation) | Variable (full name) | week | “control” (C) lines | |  | “aerobic” (A) lines | |
| --- | --- | --- | --- | --- | --- | --- | --- |
|  |  |  | comfort | CMS |  | comfort | CMS |
| N | number of individuals |  | 24 | 23 |  | 23 | 23 |
| BMavg | average body mass [g] | initial | 21.0 ± 3.3 | 20.3 ± 3.1 |  | 23.0 ± 2.8 | 23.9 ± 2.8 |
|  |  | first | 20.7 ± 3.3 | 20.5 ± 3.2 |  | 23.4 ± 2.8 | 24.3 ± 3.1 |
|  |  | second | 21.1 ± 3.4 | 20.9 ± 3.3 |  | 23.5 ± 2.8 | 24.7 ± 3.1 |
| FI | apparent food intake [g/day] | initial | 4.03 ± 0.87 | 4.10 ± 0.84 |  | 5.92 ± 0.68 | 6.40 ± 1.16 |
|  |  | first | 4.08 ± 0.84 | 4.33 ± 0.92 |  | 6.06 ± 0.88 | 6.24 ± 1.09 |
|  |  | second | 4.30 ± 0.94 | 4.24 ± 0.91 |  | 6.13 ± 1.05 | 5.78 ± 1.75 |
| FCMs | fecal corticosterone metabolites [ng/g] | initial | 70.4 ± 62.6 | 66.9 ± 67.9 |  | 50.6 ± 27.3 | 47.6 ± 20.8 |
|  |  | first | 61.7 ± 52.1 | 48.5 ± 46.1 |  | 54.5 ± 34.7 | 43.3 ± 21.1 |
|  |  | second | 65.1 ± 53.5 | 53.9 ± 49.4 |  | 53.1 ± 27.9 | 42.9 ± 21.7 |
| VO_2_swim | maximum 1‑min oxygen consumption in a swimming trial [ml O_2_/min] | initial | 3.46 ± 0.54 | 3.20 ± 0.53 |  | 5.57 ± 0.52 | 6.30 ± 0.64 |
|  |  | first | 3.40 ± 0.61 | 3.14 ± 0.65 |  | 5.49 ± 0.88 | 6.21 ± 0.56 |
|  |  | second | 3.35 ± 0.62 | 3.14 ± 0.63 |  | 5.50 ± 0.64 | 6.20 ± 0.52 |
| VO_2_avg | average oxygen consumption in a swimming trial [ml O_2_/min] | initial | 3.10 ± 0.58 | 2.81 ± 0.66 |  | 5.10 ± 0.56 | 5.89 ± 0.57 |
|  |  | first | 2.87 ± 0.71 | 2.64 ± 0.80 |  | 4.96 ± 0.97 | 5.71 ± 0.66 |
|  |  | second | 2.78 ± 0.75 | 2.68 ± 0.81 |  | 4.98 ± 0.77 | 5.74 ± 0.65 |

**Supplementary Table S3** **Likelihood ratio (LR) test statistics for significance of random effects in ANCOVA mixed models performed on scopes of deviation from baseline trait values during the experiment.** Models for body mass averaged over 4 days (BMavg), apparent daily food intake (FI), fecal corticosterone metabolites (FCMs), and measures of swimming metabolism: average oxygen consumption (VO_2_avg) and maximum 1‑min oxygen consumption (VO_2_swim). n.a. – non-applicable (factor absent from the model). s^2^ – covariance parameter estimate; SE – standard error of covariance parameter estimation; chi^2^ – difference between -2 residual log likelihoods of two models used in the LR test; p – p-value. Values of s^2^ ≤ 0 – variance estimate constrained to zero; n.a. – not applicable (tests not performed for components, where variance was constrained to zero).

| Variable (abbreviation) | Variable (full name) |  | replicate line | replicate line × sex | replicate line × treatment | replicate line × week | replicate line × treatment × week | experimental block | experimental block × test | individual |
| --- | --- | --- | --- | --- | --- | --- | --- | --- | --- | --- |
| BMavg | average body mass | s^2^(SE) | 0.00(0.00) | ≤ 0 | ≤ 0 | ≤ 0 | ≤ 0 | ≤ 0 | ≤ 0 | 0.00(0.00) |
|  |  | chi^2^ | 3.6 | n.a. | n.a. | n.a. | n.a. | n.a. | n.a. | 94.4 |
|  |  | p | 0.058 | n.a. | n.a. | n.a. | n.a. | 0.074 | n.a. | <0.001 |
| FI | apparent food intake | s^2^(SE) | 0.00(0.00) | ≤ 0 | 0.00(0.00) | ≤ 0 | ≤ 0 | ≤ 0 | ≤ 0 | 0.00(0.00) |
|  |  | chi^2^ | 5.4 | n.a. | 2 | n.a. | n.a. | n.a. | n.a. | 19.4 |
|  |  | p | 0.020 | n.a. | 0.157 | n.a. | n.a. | <0.001 | n.a. | <0.001 |
| FCMs | fecal corticosterone metabolites | s^2^(SE) | ≤ 0 | 0.00(0.00) | ≤ 0 | ≤ 0 | ≤ 0 | ≤ 0 | ≤ 0 | 0.06(0.00) |
|  |  | chi^2^ | n.a. | 2.1 | n.a. | n.a. | n.a. | n.a. | n.a. | 33.8 |
|  |  | p | n.a. | 0.147 | n.a. | n.a. | n.a. | 0.192 | n.a. | <0.001 |
| VO_2_avg | average oxygen consumption in a swimming trial | s^2^(SE) | 0.00(0.00) | 0.00(0.00) | ≤ 0 | ≤ 0 | ≤ 0 | ≤ 0 | 0.00(0.00) | 0.01(0.00) |
|  |  | chi^2^ | 11.7 | 1.3 | n.a. | n.a. | n.a. | n.a. | 3.1 | 16.6 |
|  |  | p | 0.001 | 0.254 | n.a. | n.a. | n.a. | n.a. | 0.078 | <0.001 |
| VO_2_swim | maximum 1-min oxygen consumption in a swimming trial | s^2^(SE) | 0.00(0.00) | 0.00(0.00) | ≤ 0 | ≤ 0 | ≤ 0 | ≤ 0 | 0.00(0.00) | 0.00(0.00) |
|  |  | chi^2^ | 7.6 | 3.3 | n.a. | n.a. | n.a. | n.a. | 1.3 | 10.7 |
|  |  | p | 0.006 | 0.069 | n.a. | n.a. | n.a. | 0.069 | 0.254 | 0.001 |
